# Use of Mobile Apps for Self-care in People With Parkinson Disease: Systematic Review

**DOI:** 10.2196/33944

**Published:** 2022-01-21

**Authors:** JuHee Lee, Insun Yeom, Misook L Chung, Yielin Kim, Subin Yoo, Eunyoung Kim

**Affiliations:** 1 Mo-Im Kim Nursing Research Institute, Yonsei Evidence Based Nursing Centre of Korea: A JBI Affiliated Group College of Nursing Yonsei University Seoul Republic of Korea; 2 Brain Korea 21 FOUR Project College of Nursing Yonsei University Seoul Republic of Korea; 3 College of Nursing University of Kentucky Lexington, KY United States; 4 College of Nursing Yonsei University Seoul Republic of Korea

**Keywords:** systematic review, Parkinson disease, motor symptoms, nonmotor symptoms, smartphone, mobile phone, mobile health, mobile apps, self-care, symptom, monitoring, review, disability, app, care, quality of life, self-management

## Abstract

**Background:**

Self-care is essential for people with Parkinson disease (PD) to minimize their disability and adapt to alterations in physical abilities due to this progressive neurodegenerative disorder. With rapid developments in mobile technology, many health-related mobile apps for PD have been developed and used. However, research on mobile app–based self-care in PD is insufficient.

**Objective:**

This study aimed to explore the features and characteristics of mobile apps for self-care in people with PD.

**Methods:**

This study was performed sequentially according to the PRISMA (Preferred Reporting Items for Systematic Reviews and Meta-Analyses) statement. PubMed, Embase, Cumulative Index to Nursing and Allied Health Literature, Cochrane Library, Web of Science, and PsycINFO were searched in consultation with a librarian on June 8, 2021. We used keywords including ”Parkinson disease” and ”mobile.”

**Results:**

A total of 17 studies were selected based on the inclusion criteria, including 3 randomized controlled trials and 14 observational studies or quasi-experimental studies. The use of mobile apps for self-care in people with PD focused on symptom monitoring, especially motor symptoms. Motor symptoms were objectively measured mainly through the sensors of smartphones or wearable devices and task performance. Nonmotor symptoms were monitored through task performance or self-reported questionnaires in mobile apps. Most existing studies have focused on clinical symptom assessment in people with PD, and there is a lack of studies focusing on symptom management.

**Conclusions:**

Mobile apps for people with PD have been developed and used, but strategies for self-management are insufficient. We recommend the development of mobile apps focused on self-care that can enhance symptom management and health promotion practices. Studies should also evaluate the effects of mobile apps on symptom improvement and quality of life in people with PD.

**Trial Registration:**

PROSPERO International Prospective Register of Systematic Reviews CRD42021267374; https://www.crd.york.ac.uk/prospero/display_record.php?ID=CRD42021267374.

## Introduction

The number of people with Parkinson disease (PD) has increased significantly with the aging population and rising life expectancy [1]. According to a systematic literature review that analyzed 47 studies, PD is predominantly prevalent in older adults (aged above 70 years) [2]. A study estimating life years and the prevalence of PD from 1990 to 2016 reported that the worldwide burden related to PD had more than doubled [1].

People with PD experience motor and nonmotor symptoms. Most motor symptoms include tremors, postural instability, bradykinesia, and rigidity. Nonmotor symptoms are associated with sensory abnormalities, neuropsychiatric abnormalities, sleep disorders, and autonomic dysfunction (eg, bladder, bowel, and sexual dysfunction) [3,4]. Symptom management is essential to maintain one’s functional ability, as insufficiently managed PD symptoms negatively influence quality of life and worsen physical disabilities in people with PD [5]. As defined by the theory of self-care in chronic illness, self-care in individuals with chronic diseases refers to a series of processes for maintaining health [6]. This self-care process includes detecting, interpreting, and responding to altered symptoms [6]. For effective self-care, symptom monitoring is essential to recognize changes in symptoms, along with skills to manage symptoms and perform health promotion practices [6].

Traditional interventions to improve self-care in PD have used face-to-face instruction to deliver health-promoting information, rehabilitation therapy, or interventions aiming to induce cognitive behavioral changes. Previous review studies on self-care interventions in people with PD identified interventions, most of which involved self-care management or self-care maintenance (eg, exercise, occupational therapy, health coaching, psychological strategy training, and lifestyle advice) to improve patients’ health outcomes [7,8]. All these were face-to-face interventions delivered without using mobile technology.

Mobile health (mHealth) devices have enabled improvements in diagnosis and treatment, as well as connection with distant patients [9]. Over the past few decades, dramatic advances in computer and communication technologies have led to the development of mHealth and communication technologies in the medical environment [10]. The portability and wide distribution of smartphones have enabled the development and usage of various health care apps that can track and manage symptoms, and these have strengthened self-care interventions for people with chronic illness. For example, recent systematic reviews have reported that mobile apps for type 2 diabetes that provide goal management or motivational feedback based on self-reported symptoms or vital sign monitoring are effective in reducing the fasting blood sugar and waist circumference [11,12]. In addition, a study reported that the overall survival rate of patients with advanced lung cancer improved after implementing a tracking algorithm, referred to as an “e-follow-up application,” via early relapse detection using weekly self-reports of symptoms [13].

Many mobile apps for PD patients have been developed and implemented. Moreover, 2 systematic reviews focusing on apps available in Google Play and the App Store from 2011 to 2016 found 92 and 125 apps, respectively, that were potentially useful for individuals with PD [14,15]. These reviews were conducted to identify a suitable operating system for these apps and analyze their usability and validity. However, both reviews did not provide detailed analyses regarding the use of mobile apps in self-care interventions. As there is no available curative treatment for PD, the severity of the symptoms and disease should be closely monitored to manage PD effectively. Symptom tracking using a smartphone offers the possibility of regularly monitoring patients’ symptoms over time, thereby overcoming the problem with traditional clinical assessments that provide a “snapshot” of patients’ conditions [16].

This study was performed to explore the use of mobile apps for self-care in people with PD. We specifically explored the features and characteristics of the mobile apps that were used for self-care maintenance, self-care monitoring, and self-care management.

## Methods

### Design

This study is a systematic review following the PRISMA (Preferred Reporting Items for Systematic Reviews and Meta-Analyses) 2020 statement [17]. The protocol was registered in the International Prospective Register of Systematic Reviews (Trial registration number: CRD42021267374).

### Search Strategy

The literature search was conducted in 3 steps. First, a search was conducted in PubMed using the following relevant MeSH (Medical Subject Headings) terms and free-text keywords. The term “Parkinson disease” and “mobile” were used as the keywords for the concept, and MeSH or Emtree terms linked to the search domains were used. The final search query was developed in consultation with a librarian having a PhD degree and more than 10 years of experience (see Multimedia Appendix 1). In the second step, a literature search was conducted in PubMed, Embase, Cumulative Index to Nursing and Allied Health Literature, Cochrane Library, Web of Science, and PsycINFO using the search query on June 8, 2021. All search results were reviewed by the librarian. In the last step, the references of the selected studies were manually searched by 2 researchers.

### Eligibility Criteria for the Review

The studies for the review were restricted to those related to self-care using mobile apps in adults with PD. We also included studies that were published in English from January 2003 to June 2021 in peer-reviewed journals. This start date was chosen because terms referring to phenomena such as cell phones, computers, handheld devices, and small portable wireless devices were introduced in 2003 as MeSH terms. In this study, self-care is defined as health maintenance practices, symptom tracking and monitoring, and management of symptoms [6]. Mobile apps are generally defined as computer programs or software applications for a mobile device such as a smartphone. We excluded studies that evaluated only technical issues related to mobile apps or tested them with healthy adults or those with other chronic diseases.

### Study Selection

**Figure 1:**
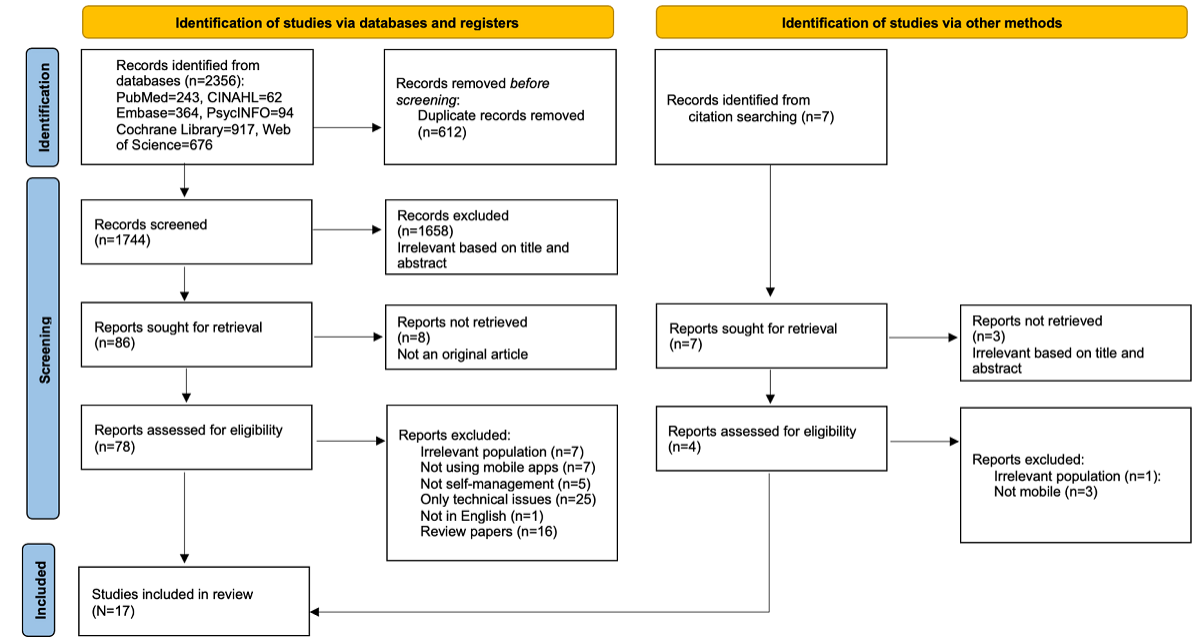
Flow diagram of the search.

All the study selection steps were initially performed by 2 researchers (EK and YK). We identified a total of 2356 studies from all databases searched in the initial stage and removed 612 duplicates. The titles and abstracts of all the remaining 1744 records were screened for potential relevance based on a standardized checklist. Of those studies, 1658 were excluded because they were considered irrelevant to the purpose of this study. In addition, 8 studies were excluded because they were not original articles, and following a full-text review, 61 studies were excluded. The reasons for exclusion were that the population did not meet the inclusion criteria, a mobile app was not used, there was no self-care context, the articles dealt with only technical issues, or they were review articles. Citation searching yielded 7 documents that were excluded as irrelevant through title, abstract, and full-text assessment. Finally, 17 studies were selected for this review, as shown in Figure 1.

### Data Extraction, Analysis, and Synthesis

Data extraction was performed independently by 2 researchers (EK and SY) using a standardized format. The following data were extracted: author(s); published year; title; published journal; country where the study was performed; aim of the study; design of the study; participants’ characteristics; the name of the mobile app; and the intervention duration, results, and limitations. For data analysis, the type of mobile app was categorized based on the method of symptom data collection and other functions. The outcome measure was categorized as satisfaction with the app, feasibility, symptom severity, and patient outcomes. The characteristics of the mobile apps were classified as self-care maintenance, self-care monitoring, and self-care management based on the theory of self-care in chronic illness [6]. Self-care maintenance was defined as health-promoting practices to maintain good health status, such as physical activity, treatment adherence, a regular sleep pattern, and nutritional intake whereas self-care monitoring was defined as tracking and recognizing symptoms leading to interpretation. Symptom monitoring was divided into monitoring of motor and nonmotor symptoms, and each symptom was classified with reference to the literature [3,4]. Self-care management pertained to behavioral changes, such as changes in the activity level, medication use, information seeking, and dietary changes. Self-care management requires symptom recognition and interpretation when physical changes occur.

### Quality Appraisal

The quality of the selected studies was assessed using tools for assessing risk of bias developed by the Cochrane Collaboration. The risk of bias in non-randomized studies of interventions (ROBINS-I) [18] was used for quality assessment of observational studies and quasi-experimental studies. The revised Cochrane risk of bias tool for randomized trials (RoB2) [19] was used for randomized controlled trials (RCTs). ROBINS-I evaluates the risk of bias in the confounding variables, selection of participants, classification of interventions, deviations from intended interventions, missing data, measurement of outcomes, selection of the reported results, and overall bias. Each section is evaluated as low, moderate, serious, critical, and no information. RoB2 consists of 6 sections, including the randomization process, deviation from the intended interventions, missing outcome data, measurement of the outcome, selection of the results, and the risk of overall bias. In each section, the risk of bias is evaluated using questions with responses “yes,” “probably yes,” “no,” “probably no,” and “no information,” and each section is finally judged as low risk, some concerns, or high risk according to the evaluation algorithm. The quality of the studies was assessed independently by 2 researchers (IY and EK). Any discrepancies were resolved by consensus.

## Results

### Study Characteristics

In total, 17 articles were analyzed in this study, as shown in Table 1. Publication years ranged from 2013 to 2020. Of the 17 selected studies, 6 were published in 2020 (35.3%). There were 12 observational studies (70.6%), 2 quasi-experimental studies (11.8%), and 3 RCTs (17.6%). The study of Gatsios et al [20] was classified as an observational study because it analyzed only the intervention group as an ancillary study of an RCT. The intervention duration varied from a single session for 30 minutes [16] to over 6 months [21-23]. More than half of the studies had intervention periods of less than 1 month [16,20,24-29]. We found that 4 studies were conducted through international collaborations in multiple countries [20,24,30,31]. Researchers in the United States conducted 7 studies, followed by England, Finland, Italy, Netherlands, and the United Kingdom with 2 studies each. Further, 1 study each was conducted in Australia, Belgium, Greece, Israel, and Scotland. A total of 1246 people with PD participated in the 17 studies. The participants’ age ranged from 34 to 84 years (mean age=63.02 years), and 58.8% (733) of the participants were male.

**Table 1:** Characteristics of the included studies.

Author (year)/country	Aim and study design	Participant characteristics (sample size, gender, age, disease duration)	App name	Frequency and duration	Results
Keränen and Liikkanen [32] (2013)/Finland	To evaluate the feasibility of medication reminders SMS; Observational	Total: 45 Male: 29 (64.4%) Age: 66.4 (SD 7.90) y	Not mentioned	4 weeks	Most were satisfied with usability (69%). The majority wanted to continue using the system (80%).
Pan et al [28] (2015)/United States	To develop and test a mobile app to assess motor symptom severity; Observational	Total: 40 Male: 35 (87.5%) Age: 68.5 (SD 9.5) y Disease duration: 6.6 (SD 9.5) y	PD^a^ Dr	A single motor performance test session	PD Dr could effectively detect hand resting tremor and gait difficulty and estimate motor symptom severity using the captured motion features.
Kassavetis et al [16] (2015)/Unit ed Kingdom	To develop and test stand-alone software for smartphones to assess motor symptoms in PD patients; Observational	Total: 14 Male: 7 (50%) Age: 54.7 (range 34-75) y Disease duration: 3.7 (SD 2.0) y	Not mentioned	A single motor performance test session for 30 minutes	Symptom severity could be assessed from the motion data (tremor, bradykinesia).
Lee et al [29] (2016)/Australia	To generate a predictive model for motor symptom severity using captured data and to evaluate compliance and user satisfaction in a smartphone app; Observational	Total: 103 Male: 52 (50.5%) Age: 66.5 (range 38-91) y Disease duration: 8.75 (range 0.5- 24) y	Not mentioned	Twice within 2 weeks	Symptom severity could be assessed from the motion data (tremor, bradykinesia, cognition). A prediction model accounted for 52.3% of the variation in motor symptoms. Participants showed high compliance (96%). Most are satisfied with usability (83%) and usefulness (97%).
Silva de Lima et al [33] (2018)/Netherlands	To assess the relationship between the severity of motor fluctuation and walking time collected using a mobile app; Observational	Total: 304 Male: 164 (54%) Age: 63.1 (SD 8.5) y Disease duration: 6.1 (SD 4.3) y	The Fox Wearable Companion app	24 hours for 13 weeks	Mean walking time was related to the severity of motor symptoms. The postmedication activity was on average higher than the premedication activity.
Zhan et al [21] (2018)/United States	To develop an objective measurement tool (mPDS^b^) to assess PD severity; Observational	Total: 169 (129 PD, 23 clinics with PD, 17 clinics without PD) Age: 58.7 (SD 8.6), 64.6 (SD 11.5), and 54.2 (SD 16.5) y Disease duration: 4.3 (SD 4.4) y, 7.0 (SD 4.1) y, and N/A^c^	HopkinsPD	3 times for 6 months	For mPDS generation, 5 activities were selected (gait, balance, finger tapping, voice, and reaction time). The mPDS detected intraday symptom fluctuations. Motor symptom severity could be estimated from mPDS.
Elm et al [22] (2019)/United States	To evaluate the feasibility of a clinician dashboard to monitor patient symptoms through data collected from ePROs^c^ and a smart watch; Observational	Total: 39 Male: 29 (74%) Age: 61.9 (SD 10.5) y Disease duration: 7.1 (SD 4.8) y	Fox Wearable Companion app	3 times for 6 months	Participants’ compliance rate was 66%. Medication compliance and the severity of ePRO symptoms from the dashboard were the most beneficial components for clinicians’ decisions.
Gatsios et al [20] (2020)/Italy, Greece, England	To evaluate the validity and clinical usefulness of data collected using a smartphone and wearable device; Observational	Total: 75 Male: 43 (60%) Age: 67.7 (SD 8.7) y Disease duration: 9.2 (SD 4.4) y	PD manager	12 hours for 11-14 days	Participants’ compliance rate was 87%. Collected data from PD manager effectively detected the tremor.
Habets et al [26] (2020)/Netherlands	To evaluate the validity of the eDiary app to collect data using the EMA^d^ method; Observational	Total: 20 Male: 16 (80%) Age: 63 (SD 7) y Disease duration: 8 (SD 6) y	Not mentioned	7 times per day for 14 days	eDiary using EMA effectively captured the relationship between affect, motor performance, and motor symptoms.
Landers and Ellis [34](2020)/United States	To explore the feasibility, safety, and effectiveness of an exercise program to promote physical activity using a mobile app; Observational	Total: 28 Male: 6 (21.4%) Age: 62.1 (SD 9.6) y Disease duration: 3.3 (SD 2.5) y	9zest Parkinson’s Therapy	30-60 minutes, 3-5 times per week for 12 weeks for at least 150 minutes per week	Complete compliance was found in 42.9% of participants, and a majority were satisfied with the app exercise (89.5%). Significant improvement was observed in the PDQ8^e^ scores, TUG test^f^, and STS test^g^ after 8 weeks.
Motolese et al [25] (2020)/Italy	To evaluate the feasibility of remote patient monitoring using a smartphone; Observational	Total: 54 Male: 36 (67%) Age: 66.5 (range 59.7-72.2) y Disease duration: 6.5 (range 4-11) y	EncephaLog Home	At least 2 times per week for 3 weeks	Completed compliance was 29.6%. Motor symptom severity could be estimated from the captured motion data (gait, tapping, tremor, and cognition).
Wu and Cronin-Golomb [27] (2020)/United States	To investigate the relationship between sleep quality and daytime functioning based on data collected using EMA and actigraphy; Observational	Total: 20 Male: 13 (65%) Age: 66.5 (SD 9.3) y Disease duration: 6.0 (SD 4.3) y	SymTrend	Every day over 2 weeks	The compliance rate was 91%-94%. Subjective sleep quality significantly predicted next-day anxiety. Other variables were not related to each other.
Horin et al [35](2019)/United States	To evaluate the usability of a mobile app to improve motor symptoms (gait, speech, and dexterity); Quasi-experimental	Total: 37 (I^h^: 17, C^i^: 20) Male: 22 (60%, I), 26 (70%, C) Age: 63.4 (SD 8.6) y (I), 64.9 (SD 8.4) y (C) Disease duration: 6.7 (SD 5.6) y (I), 6.0 (SD 4.3) y (C)	Beats Medical Parkinson’s Treatment App	30-60 minutes, once a day for 90 days	Compliance was moderate (64.6%-67.4%). There were no significant improvements in gait, speech, or dexterity.
Kuosmanen et al [24] (2020)/Finland, United Kingdom	To monitor and evaluate hand tremors using a smartphone game and assess medication effects on hand tremors; Quasi-experimental	Total: 13 Male: 5 (38.5%) Age: 64.7 (SD 6.8) y Disease duration: 7.1 (range 2-17) y	STOP (the Sentient Tracking of Parkinson’s) app	For 1 month	Motor symptom severity was estimated from the collected tremor data. Through the collected accelerometer signals, the medication effect on rigidity and bradykinesia was confirmed.
Ginis et al [31] (2016)/Belgium, Israel	To compare the effects of gait training using a mobile app and conventional home-based training; RCT^j^ (pilot)	Total: 38 (I: 22, C: 18) Male: 6 (15%, I), 11 (27.8%, C) Age: 67.3 (SD 8.1) y (I), 66.1 (SD 8.1) y (C) Disease duration: 10.7 (SD 5.4) y (I), 11.7 (SD 7.6) y (C)	CuPiD system	30 minutes, at least 3 times per week for 6 weeks, with weekly home visits by the researcher	Both groups showed significant improvements in gait speed. The CuPiD group improved significantly more in balance than the control group.
Lakshminarayana et al [30] (2017)/England, Scotland	To evaluate the effectiveness of mobile apps in monitoring PD symptoms; RCT	Total: 201 (I: 94, C: 107) Male: 128 (63.8%, I), 116 (57.9%, C) Age: 59.9 (SD 9.2) y (I), 60.7 (SD 10.3) y (C) Disease duration: 5.7 (SD 4.2) y (I), 5.5 (SD 4.9) y (C)	PTA (the Parkinson’s Tracker App)	Once per day or every other day for 16 weeks	The PTA group reported an improvement in medication adherence and PCQ-PD^k^ compared with TAU^l^.
Ellis et al [23] (2019)/United States	To evaluate the safety and effectiveness of an exercise program using the mobile app; RCT (single-blind, pilot)	Total: 44 (I: 23, C: 21) Male: 25 (57.7%, I), 23 (52%, C) Age: 64.8 (SD 8.5) y (I), 63.3 (SD 10.6) y (C) Disease duration: 5.9 (SD 3.5) y (I), 3.7 (SD 2.1) y (C)	Wellpepper	5-7 times or at least 3 times per week for 6 months and later extended to 12 months	Daily steps and 6MWT^m^ did not show statistically significant between-group differences. PDQ-39^n^ improved in the mobile app group.

^a^PD: Parkinson disease.

^b^mPDS: mobile Parkinson disease score.

^c^N/A: not available.

^d^EMA: ecological momentary assessment.

^e^PDQ8: Parkinson Disease Questionnaire 8.

^f^TUG test: timed up-and-go test.

^g^STS test: sit-to-stand test.

^h^I: intervention group.

^i^C: control group.

^j^RCT: randomized controlled trial.

^k^PCQ-PD: Patient-Centered Questionnaire for Parkinson Disease.

^l^TAU: treatment as usual.

^m^6MWT: 6-meter walking test.

^n^PDQ-39: Parkinson Disease Quality of Life.

### Quality Appraisal

The quality appraisal results of the 17 selected studies are as follows. In 14 observational studies and quasi-experimental studies, there was no high risk of bias in terms of the confounding variables, classification of interventions, deviations from intended interventions, missing data, or measurement of outcomes. Among the 14 studies, 1 was evaluated as having “serious” concerns regarding the selection of participants and “critical” concerns for the selection of the reported results [21]. Furthermore, 2 studies were evaluated as having “serious” concerns regarding the selection of participants and the reported results [24,35]. Thus, these 3 studies were evaluated as having “serious” or “critical” concerns in at least 1 of the 7 domains in ROBINS-I, as observed in Table 2. This review was conducted to explore the use of mobile apps in PD and focus on the features and characteristics of these apps, and not to evaluate the effectiveness of interventions. Therefore, 3 studies evaluated as “critical” and “serious” were included in the analysis to determine the usage characteristics of the mobile apps.

RoB2 was used to appraise 3 RCTs of which 2 reported only the baseline characteristics of participants without a prior homogeneity analysis between the intervention and control groups [23,31]. However, these studies reported a computer-generated stratified randomization procedure in the randomization process. Therefore, they were considered as having “low risk” in the randomization process and “low risk” in all the other domains of RoB2. The other study was also deemed to be “low risk” in all the domains of RoB2 [30]. All RCTs were evaluated as having a low risk of bias, as observed in Table 3.

**Table 2:** Quality appraisal of the studies: risk of bias in nonrandomized studies of interventions.

Study (year)	Confounding	Participant selection	Intervention classification	Deviations from intended interventions	Missing data	Outcome measurements	Selection of the reported results	Overall
Keränen and Liikkanen [32] (2013)	Low	Moderate	Low	Low	Low	Low	Low	Moderate
Pan et al [28] (2015)	Low	Moderate	Moderate	Low	Low	Low	Low	Moderate
Kassavetis et al [16] (2015)	Low	Low	Low	Low	Low	Low	Low	Low
Lee et al [29] (2016)	Low	Low	Moderate	Low	Low	Low	Low	Moderate
Silva de Lima et al [33] (2018)	Low	Low	Low	Low	Low	Low	Low	Low
Zhan et al [21] (2018)	Low	Serious	Low	NI^a^	Low	Low	Critical	Critical
Elm et al [22] (2019)	Low	Low	Low	Moderate	Low	Low	Low	Moderate
Gatsios et al [20] (2020)	Low	Low	Low	Low	Low	Low	Low	Low
Habets et al [26] (2020)	Low	Low	Low	Low	Low	Low	Low	Low
Landers and Ellis [34] (2020)	Low	Moderate	Low	Low	Low	Low	Low	Moderate
Motolese et al [25] (2020)	Low	Low	Low	Low	Low	Low	Low	Low
Wu and Cronin-Golomb [27] (2020)	Low	Low	Low	Low	Low	Low	Low	Low
Horin et al [35] (2019)	Low	Low	Low	Low	NI	Low	Serious	Serious
Kuosmanenet al [24] (2020)	Low	Serious	Moderate	Moderate	Low	Low	Low	Serious

^a^NI: no information.

**Table 3:** Quality appraisal of the studies: revised Cochrane risk of bias tool for randomized trials.

Author (year)	Randomization process	Deviations from intended interventions	Missing outcome data	Outcome measurements	Selection of the reported results	Overall
Ginis et al [31] (2016)	Low risk	Low risk	Low risk	Low risk	Low risk	Low risk
Lakshminarayana et al [30] (2017)	Low risk	Low risk	Low risk	Low risk	Low risk	Low risk
Ellis et al [23] (2019)	Low risk	Low risk	Low risk	Low risk	Low risk	Low risk

### Features and Usage of the Mobile Apps

The mobile app system configurations used in this review included 5 types of symptom data collection, reminder, or user interaction functions, given in Table 4. Types of symptom data collection included using the sensor of a smartphone or wearable device, task performance, voice recordings, and self-reported surveys. Among 17 studies, 6 studies collected symptoms using a smartphone accelerometer and gyroscope [16,20,21,24,25,28]. Further, 7 studies used wearable devices [20,23,26,27,31,33,35], which included a smartwatch [20,33], a smart insole [20], an actigraph such as a Fitbit [23], and sensors attached to the ankle [31,35], chest [26], or wrist [26]. Task performance was assessed in 9 studies [16,20,21,24,25,29,30,34,35]. Finger tapping was the most common with 5 studies using it [16,21,25,29,30], followed by cognitive function tests using games or memory tests in 4 studies [20,25,29,30]. There were games such as a ball game [24] and a 9-hole peg game [35] for motor symptom measurement. Task performance also included the sit-to-stand test [34] and the timed up-and-go test [25,34]. Voice data were collected using the microphone of a smartphone in 2 studies [20,21], and 1 study collected voice data using a head-mounted condenser microphone [35]. Another method of collecting data on symptoms was a self-reported survey [20,22,24,26,27,30,34]. Structured survey tools for electronic patient-reported outcomes [22] and ecological momentary assessments (EMAs) [26,27] were developed. EMAs collect subjective experiences at multiple semirandomized moments during the day to better capture symptom changes.

Functions other than symptom collection were reminders [22,24,30,32,33] or user interactions [23,28,31,34]. Reminder functions, such as symptomatic alerts and medication reminders, were the most common features to assist people with PD in self-care. The user interaction functions provided feedback based on patient activity [23,31,34] or communication with a medical care facility server [28].

The measured outcomes of mobile app usage were participants’ satisfaction with the mobile app [25,29,32,34], compliance with using the app [20,22,23,25,27,29,31,34,35], and correlations between the collected symptom data and symptom severity for people with PD [16,20-22,24,26-29,33] (Table 4). Satisfaction with the mobile app was investigated using structured items in various studies. The overall satisfaction rate was 83% to 89.5% [25,29,34], and 1 study reported a rate of 69% [32]. In 1 study, 80% of the users were willing to use the app again because it provided medication reminders via SMS [32], and 97% of the users who used the app to measure motor symptoms responded that the app was useful [29]. Compliance mostly ranged from relatively high (87% to 96%) [20,27,29] to moderate (42.9% to 67.4%) [22,34,35], whereas 1 study reported very low compliance (29.6%) [25]. A study that compared groups with and without a mobile intervention reported no between-group difference in compliance [23]. Several studies reported that the data collected through the app could be used to estimate the severity of motor symptoms [16,21,24,28,29,33].

Patient outcomes were measured in 5 studies. The measured patient outcomes were changes in symptoms or activity levels [23,30,31,34,35], medication adherence [30], and quality of life [23,30,31,34]. Studies have reported an improvement in patient symptoms, activity levels, and gait balance in the mobile app group [31,34]. Further, 2 studies compared activity-level differences between groups using mobile apps and usual interventions; however, there were no differences between the 2 groups in terms of symptoms or activity levels [23,30]. Several studies provided medication reminders using apps, but only 1 study measured medication adherence. This study reported that medication reminders sent using apps led to improved medication adherence [30]. Some studies that measured quality of life reported improvement [23,34], but others did not [30,31].

**Table 4:** Features and usage of the mobile apps in the included studies.

Study (year)	Features of the mobile app										Outcome measurements						
	Type of symptom data collection						Function			Satisfaction			Feasibility		Symptom severity		Patient outcomes
	Smartphone sensor	Task performance	Voice data	Wearable device	Self-report	Reminder		User interaction									
Keränen and Liikkanen [32] (2013)						✓ SMS			✓								
Pan et al [28] (2015)	✓							✓						✓			
Kassavetis et al [16] (2015)	✓	✓												✓			
Lee et al [29] (2016)		✓ CIT^a^							✓			✓		✓			
Silva de Lima et al [33] (2018)				✓		✓								✓			
Zhan et al [21] (2018)	✓	✓	✓											✓ mPDS^b^			
Elm et al [22] (2019)					✓ ePROs^c^	✓						✓		✓			
Gatsios et al [20] (2020)	✓	✓	✓	✓	✓							✓		✓			
Habets et al [26] (2020)				✓	✓ EMA^d^									✓			
Landers and Ellis [34] (2020)		✓			✓			✓	✓			✓				✓	
Motolese et al [25] (2020)	✓	✓ CIT							✓			✓					
Wu and Cronin-Golomb [27] (2020)				✓	✓ EMA							✓		✓			
Horin et al [35] (2019)		✓ Game	✓	✓								✓				✓	
Kuosmanen et al [24] (2020)	✓	✓ Game			✓	✓								✓			
Ginis et al [31] (2016)				✓				✓				✓				✓	
Lakshminarayana et al [30] (2017)		✓ Game			✓	✓										✓	
Ellis et al [23] (2019)				✓				✓				✓				✓	

^a^CIT: cognitive interference test.

^b^mPDS: mobile Parkinson disease score.

^c^ePROs: electronic patient-reported outcomes.

^d^EMA: ecological momentary assessment.

### Self-care Maintenance

The use of mobile apps for self-care maintenance in this review encompassed medication adherence and physical activity, as indicated in Table 5. Among the 17 studies, 6 were related to medication [22,24,26,30,32,33]. These included 1 RCT [30], 1 quasi-experimental study [24], and 4 observational studies. Of these, 5 studies provided medication reminders via SMS [32] or web push notifications in the apps [22,24,30,33] to promote medication adherence according to a preset medication time. Studies using web push notifications also recorded medication tracking through responses to medication reminders. Another study collected data on medication intake through EMAs [26]. As outcome measures, studies evaluated medication adherence using self-report questionnaires, participants’ satisfaction, as well the relationship between symptom fluctuations or severity and medication intake [24,26,30,32,33]. Another study provided notifications to promote medication adherence through a mobile app, but it did not measure the relevant outcomes [22].

Physical activity was measured in 3 studies among which 2 studies provided tailored exercises to each participant through a mobile app [23,34], and another study consisted of an exercise program for 30 minutes to improve gait, speech, and dexterity symptoms [35]. There was an observational study [34], a quasi-experimental study [35], and an RCT [23]. Landers and Ellis [34] provided tailored video-guided exercises using a proprietary algorithm based on motor symptom data collected through the app. Ellis et al [23] compared the delivery of a prescribed set of exercises with and without mHealth technology. All studies collected information on motor symptoms to measure symptom- and activity-level changes and evaluated the feasibility of the mobile apps based on compliance. Patient outcomes such as quality of life were evaluated in 2 studies [23,34].

**Table 5:** Self-management characteristics of the mobile apps.

Authors (year)	Self-care maintenance			Self-care monitoring													Self-care management	
			Motor symptoms							Nonmotor symptoms								
	PA^a^	TA^b^	Tr.^c^		Rig.^d^	BK^e^	PI^f^	Others	SA^g^		NS^h^	SD^i^	AD^j^	Others				
Keränen and Liikkanen [32] (2013)		✓																
Pan et al [28] (2015)			✓				✓											
Kassavetis et al [16] (2015)			✓			✓												
Lee et al [29] (2016)			✓			✓					✓ Cognition							
Silva de Lima et al [33] (2018)		✓						✓ PA level										
Zhan et al [21] (2018)						✓	✓ Gait	✓ Speech										
Elm et al [22] (2019)		✓	✓		✓	✓	✓ Gait	✓ Speech					✓ Constipation					
Gatsios et al [20] (2020)			✓			✓	✓	✓ Speech/PA level			✓ Cognition/emotion	✓						
Habets et al [26] (2020)		✓	✓		✓	✓	✓	✓ Speech/ PA level	✓ Hallucinations		✓ Emotion	✓		✓ Fatigue				
Landers and Ellis [34] (2020)	✓		✓				✓	✓ Fall/PA level										
Motolese et al [25] (2020)			✓			✓	✓ Gait				✓ Cognition							
Wu and Cronin-Golomb [27] (2020)											✓ Cognition/emotion	✓		✓ Fatigue				
Horin et al [35] (2019)	✓ Gait, speech, dexterity		✓			✓	✓ Gait	✓ Speech										
Kuosmanen et al [24] (2020)		✓	✓					✓ Dyskinesia										
Ginis et al [31] (2016)							✓ Gait								✓ Gait training			
Lakshminarayana et al [30] (2017)		✓				✓		✓ PA level			✓ Cognition/emotion	✓		✓ Pain				
Ellis et al [23] (2019)	✓							✓ PA level										

^a^PA: physical activity.

^b^TA: treatment adherence.

^c^Tr.: tremor.

^d^Rig.: rigidity.

^e^BK: bradykinesia.

^f^PI: postural instability.

^g^SA: sensory abnormalities.

^h^NS: neuropsychiatric symptoms.

^i^SD: sleep disorder.

^j^AD: autonomic dysfunction.

### Self-care Monitoring

Symptoms were monitored in 16 studies. Among them, 7 studies involved self-care monitoring (ie, without self-care maintenance or self-care management) (Table 5). Self-care monitoring assessed the motor and nonmotor symptoms of PD. The most frequently monitored motor symptom was tremor [16,20,22,24-26,28,29,34,35], followed by bradykinesia [16,20-22,25,26,29,30,35], and postural instability and gait [20-22,25,26,28,31,34,35]. Data on rigidity were collected in 2 studies [22,26]. In addition to typical motor symptoms, speech [20-22,26,35], physical activity [20,23,26,30,33,34], and dyskinesia [24] were monitored. Although not technically a motor symptom, fall events [34] were also monitored. Different methods were used to monitor each motor symptom. Smartphones or wearable accelerometers and gyroscopes were mainly used to collect data on tremor [16,20,24-26,28,35], postural instability, and gait symptoms [20,21,28,31,35]. Bradykinesia was usually assessed using task performance such as finger tapping on the screen [16,21,25,29,30], or a 9-hole peg game designed to arouse the patients’ interest [35]. Postural instability and tremor were also monitored through performance tasks. Postural instability was assessed by having participants perform the sit-to-stand test [34] and the timed up-and-go test [25,34]. Tremor data were collected using a ball game [24] or rapid alternating movements of the hand holding a smartphone [29]. Rigidity was assessed using self-reported questionnaires only [22,26]. Symptoms related to speech were assessed by self-reports on the severity of symptoms [22,26] or by collecting voice data using a smartphone’s microphone or a head-mounted condenser microphone and a digital recorder [20,21,35]. Fall event and dyskinesia data were collected through self-reports. The physical activity level was assessed using self-report questionnaires [26,30,34] or wearable devices [20,23,33].

Among the 7 studies involving self-care monitoring of nonmotor symptoms, neuropsychiatric symptoms (eg, those related to cognition or emotion) were the most common, appearing in 6 studies [20,25-27,29,30]. Symptoms related to sleep disorders were tracked in 4 studies [20,26,27,30]. Other studies gathered information on fatigue [26,27], constipation [22], hallucinations [26], and pain [30]. All nonmotor symptom data were collected using self-reporting questionnaires, except for data on sleep symptoms and cognitive symptoms, which were investigated objectively using wearable devices and task performance, respectively. Sleep data, such as sleep duration and wakefulness, were automatically collected through wearable devices, such as actigraphs [27] or smart watches [20]. Cognition data were collected using task performance, such as cognitive interference tests, memory tests, and cognitive games [20,25,29,30].

Outcomes in self-care monitoring included motor symptom severity estimation from the mobile app data. The severity of symptoms was evaluated in comparison with the clinical scales used in PD such as the Unified Parkinson’s Disease Rating Scale. Tremor was the most frequently assessed symptom [16,20,24,28,29], followed by bradykinesia [16,21,29]. The mobile Parkinson disease score and ePROs were developed to measure motor symptoms through the mobile apps [21,22]. The results were compared with clinical data such as the Unified Parkinson’s Disease Rating Scale.

### Self-care Management

There was 1 study related to self-care management that conducted an RCT with a gait symptom improvement program [31]. The study participants performed walking exercises at least 3 times a week for 30 minutes according to the researchers’ instructions. The intervention group members were additionally provided audio biofeedback to improve their balance, gait speed, stride length, and cadence based on the symptoms collected through the sensors on their ankles. This study assessed endurance and quality of life to compare the effectiveness of the gait improvement program with that of conventional gait training.

## Discussion

### Principal Findings

This review aimed to explore the types, characteristics, and outcomes of mobile apps for self-care in people with PD. Even though mHealth apps have been used widely and positive awareness has grown in the past several years [36], only 17 studies were confirmed as novel studies in the present review. This suggests that the usage of mobile apps for self-care by people with PD is in the early stage. Most studies were observational, whereas a few studies investigated the effects of mobile apps on self-care. There were 3 RCTs, which are insufficient to evaluate the effectiveness of mobile apps used for self-care in people with PD. Most studies investigated self-care monitoring, followed by self-care maintenance and self-care management. These results suggest that the usage of mobile apps for self-care in people with PD is focused on self-care monitoring. Self-care monitoring is important to provide a direction for self-care maintenance and management behaviors in people with PD [6]. Self-care refers to self-monitoring of symptom changes and a series of processes for maintaining a healthy life. Self-care monitoring must be accompanied by health-promoting behaviors and responses to changes in symptoms [6]. However, almost half of the studies focused only on self-care monitoring [16,20,21,25,27-29].

### Features and Usage of the Mobile Apps

Self-care mobile apps for people with other chronic illnesses focused on medication reminders, patient-provider communication, data collection, and transfers of patient outcomes [37]. Specialized software programs or applications were used to check symptoms, connect with patients and diabetes educators in real time, or record a food diary; studies have also deployed wireless or Bluetooth-compatible devices to transfer data automatically from blood pressure monitors, blood glucose meters, electrocardiograms, and scales [37]. Mobile apps for PD use specialized software or applications to generate medication reminders, track symptom data, and facilitate communication between patients and medical care facility servers. However, the most notable mobile apps for people with PD involve using the sensors of smartphones or wearable devices. Accelerometers and gyroscopes of smartphones or wearable devices have advanced from a technological standpoint in that they can effectively capture tremors, postural instability changes, and minute differences in the positions of people with PD [20,28,35]. Studies have used smartwatches or actigraphy to automatically collect sleep data in people with PD [20,27]. According to a qualitative study examining users’ perceptions of mHealth apps, many participants preferred tracking technologies based on sensors, such as accelerometers and gyroscopes [36]. Data collection based on sensors or task performance can partially solve the problem of unreliable self-reported data in tracing. Compared to the sensors of smartphones or wearables that would automatically collect data, performance tasks or self-reported questionnaires require the patient to input information directly. Manually inputting data takes time and effort, which could decrease compliance with app usage. However, some symptoms can be monitored only through performance tasks or self-reporting.

Most studies in this review measured adherence to mobile apps, which can be linked to clinical symptom assessment in people with PD. Compliance is an important technology-related issue for interventions using mobile apps. The study with the lowest compliance reported that participants dropped out due to difficulties using smartphones, clinical symptoms, or lack of time [25]. Digital literacy was a factor associated with the use of mobile apps [38]. People with a lower socioeconomic status and those who were older had low awareness of health apps or faced difficulties in using them [36]. A study in this review reported that motor-related aspects of daily living, patients’ self-rated health status, and caregivers’ burden were the determinants of compliance [20]. These factors could be barriers hindering continued app usage. Elm et al [22] reported declining amounts of streaming and reporting over time, specifically after the first 3 months. As a study pointed out, patients preferred straightforward and simple methods [36]. People with PD might experience difficulties using a smartphone because they are older and have motor symptoms. User-centered interface configurations, which consider the characteristics such as the age and disease of the users, should be considered to increase compliance.

PD involves various motor symptoms due to a marked decrease in the neurotransmitter dopamine, which needs accurate assessment of disease-related symptoms [4]. The studies included in this review showed that data collected through mobile apps could effectively assess disease severity in people with PD. This finding suggests the possibility of regular home-based assessments to capture symptom changes between follow-up visits with clinicians.

The goal of self-care in chronic illness is to maintain optimal living with the disease, which means maintaining one’s health status, improving well-being and quality of life, reducing health care use, and decreasing mortality and symptom burden [6]. It is necessary to assess the clinical outcomes related to self-care to evaluate the effects of using mobile apps for self-care. In this regard, 3 systematic reviews about self-care apps for people with chronic illnesses (ie, chronic lung disease, cardiovascular disease, and diabetes mellitus) identified effectiveness in terms of clinical outcomes such as changes in physical function and clinical results (eg, 6-minute walking test, hemoglobin A_1c_, blood pressure, blood glucose, or body weight), compliance with a treatment regimen, performance of self-care tasks, and quality of life [11,37,38]. Among the studies considered in this review, 5 assessed clinical outcomes related to self-care. The results of these studies showed that the usage of mobile apps in patients with PD was still insufficient to confirm whether patient outcomes such as changes in symptoms or activity levels, medication adherence, and quality of life had improved.

### Self-care Maintenance

It is known that the motor symptoms of PD can be effectively controlled by medications [4]; therefore, medication adherence is very important in PD. It is not surprising that the first study on mobile apps for self-care in PD involved medication reminders to promote medication adherence [32]. Web push notifications are effective in tracking medication adherence, whereas SMS can only provide medication reminders. Recording responses to medication reminders is a more objective method for assessing medication adherence than a self-reporting questionnaire. However, no studies analyzed collected medication records to assess medication adherence. This finding suggests that future research needs to focus on symptom changes according to medication adherence rather than subjectively measuring adherence.

Physical activity has been established as the most effective way of improving physical and cognitive functions in people with PD [39]. Many PD patients struggle to participate in exercise programs due to their functional limitations and abilities [34]. They may sometimes be motivated to perform healthy behaviors but may not know the right way to perform them [36]. Many people using health-promoting apps value personalized and tailored information [36]. People with PD need personalized coaching and specific exercise planning programs tailored to their functional abilities. A study found that a customized exercise program using a mobile app could be safely and effectively provided to people with PD who could not regularly participate in exercise programs due to symptoms or functional changes [34]. Various face-to-face interventions focused on improving fatigue, stress, sleep, and nutrition were provided to maintain a healthy lifestyle via self-management [7,8]. However, the interventions using mobile apps focused mostly on medication adherence and physical activity.

### Self-care Monitoring

Among the motor symptoms, tremor, bradykinesia, postural instability, and gait were monitored frequently. The results show that monitoring these symptoms has important implications for the management of PD. Rigidity, which is referred to as a major motor symptom in the literature, was assessed less frequently than other symptoms [4]. A reason for this might be that rigidity can only be measured through self-reporting, unlike symptoms such as tremor, bradykinesia, postural instability, and gait, which can be objectively measured through wearable devices or task performance.

People with PD experience various nonmotor symptoms in addition to motor symptoms [3,4]. Similar to motor symptoms, nonmotor symptoms contribute toward deteriorating quality of life [5]. This review found that self-care monitoring using a mobile app in people with PD often focused more on monitoring motor symptoms than nonmotor symptoms. The nonmotor symptoms experienced by people with PD include cognitive impairment, sleep problems, urinary problems, pain, fatigue, and constipation [5]. This review showed that cognitive or emotional impairment and sleep were the main nonmotor symptoms monitored using mobile apps. Except for cognitive impairment and sleep disturbance, other nonmotor symptoms are subjective and difficult to assess. As nonmotor symptoms have a significant impact on the quality of life of patients with PD, they should be monitored using various structured tools.

### Self-care Management

Previous studies reported interventions applied for self-care management in people with diabetes mellitus or hypertension, such as goal management, motivational feedback, and health coaching through mobile apps [11,12]. These interventions have been confirmed in face-to-face interventions for self-care management. Only 1 study analyzed a self-care management intervention through a mobile app for people with PD. The study involved gait training with audio biofeedback [31]. Because this app provided feedback according to the individual's gait performance, it had a corrective effect on gait symptoms. Self-care management interventions function as a navigator to change health practices or seek medical resources in a timely manner when the symptoms occur. This review confirms that self-care management interventions using mobile apps in people with PD are highly insufficient. There is a need to develop mobile apps for patients with PD that can guide medication adherence, physical activity enhancement, or use of health care resources when symptom changes occur.

### Strengths and Limitations

Several reviews on mobile apps for people with PD have been conducted. However, previous reviews compared the iOS and Android operating systems or analyzed the potential usability of these apps for assessing and treating PD [14,15]. In contrast, we focused on analyzing the usage of mobile apps for self-care. As PD is a progressive disease, self-care is very important for maintenance, monitoring, and symptom management. This review makes a meaningful contribution to existing research by identifying the strengths and weaknesses related to the usage and development of mobile apps for self-care in people with PD. Nevertheless, several limitations should be noted. First, owing to the low number of RCTs, we could not compare the effectiveness of mobile apps for self-care. Second, because we excluded protocols, studies limited to only technical issues, and articles published in non-English languages, there was a potential bias in literature selection that could have influenced the interpretation of the results.

### Implications

We found that the motor and nonmotor symptoms of patients with PD could be continuously monitored through mobile apps and that disease severity could be estimated using the collected data. Smartphone sensors and wearable devices measured motor symptoms objectively. A structured tool could be a possible option to collect nonmotor symptom data. Studies on mobile apps for patients with PD showed that interventions targeting medication adherence or physical activity were applicable. There is a need to develop self-care interventions that organically connect health promotion behaviors, symptom monitoring, and behavior changes with the usage of mobile apps in patients with PD.

### Conclusions

This review identified that the usage of mobile apps for self-care in people with PD focused only on disease-specific characteristics and did not involve approaches to symptom management. These results imply that future research on mobile app development for people with PD should involve strategies for self-care management and maintenance based on symptom monitoring. Further research is needed to build evidence to support the usage of mobile apps for self-care in people with PD and evaluate the effects of such apps on quality of life and symptom improvement.

## Appendix

Multimedia Appendix 1 Searching terms.

## Abbreviations

EMAs: ecological momentary assessments
mHealth: mobile health
PD: Parkinson disease
RCTs: randomized controlled trials
ROBINS-I: risk of bias in non-randomized studies of interventions
RoB2: revised Cochrane risk of bias tool for randomized trials
