# Supplementary material for: Use of Mobile Apps for Self-care in People With Parkinson Disease: Systematic Review
Source: JMIR Mhealth Uhealth. 2022 Jan 21;10(1):e33944. doi: 10.2196/33944 (PMC8817212; doi:10.2196/33944)
Supplement: Multimedia Appendix 1 [file mhealth_v10i1e33944_app1.docx]

**Table S1. Searching Terms**

|  | Parkinson’s disease | Mobile |
| --- | --- | --- |
| Keywords | “Parkinson Disease” OR “Parkinson Disease” OR “Parkinsonism” OR “Primary Parkinsonism” OR “Paralysis Agitans” OR “Parkinsonian Disorder” OR “Parkinsonian Disorders” OR “Parkinson*” OR “PD” | “Cell Phone” OR “Cell Phones” OR “cellular phone” OR “cellular phones” OR “cellular phone” OR “Smartphone” OR “Smart-phone” OR “Smartphones” OR “Smart-phones” OR “mobile phone” OR “mobile phone  S” OR “mobile phones” OR “smart device” OR “smart devices” OR “m-health” OR “mobile health” OR “Mobile Clinics” OR “tablet devices” OR “tablet device” OR “tablet-based” OR “tablet PC” OR “mobile-based” OR “Mobile Applications” |
| MeSH | “Parkinson Disease” OR “Parkinsonian Disorders” OR “Paralysis Agitans” | “Cell Phone” OR “Computers, Handheld” OR “Smartphone” OR “Mobile Applications” |
| Emtree | “Parkinson Disease” OR “Parkinsonism” | “mobile phone” OR “personal digital assistant” OR “smartphone” OR “mobile application” |
| CINAHLMH subject heading | “Parkinson Disease” OR “Parkinsonian Disorders” | “Cell Phone” OR “Computers, Hand-Held” OR “Smartphone” OR “Mobile Applications” |
| PsycINFO | “Parkinson Disease” OR “Parkinsonism” | “mobile phones” OR “Smartphones” OR “Smartphone Use” |
